# *Rhizobium tumorigenes* sp. nov., a novel plant tumorigenic bacterium isolated from cane gall tumors on thornless blackberry

**DOI:** 10.1038/s41598-018-27485-z

**Published:** 2018-06-13

**Authors:** Nemanja Kuzmanović, Kornelia Smalla, Sabine Gronow, Joanna Puławska

**Affiliations:** 1 Julius Kühn-Institut, Federal Research Centre for Cultivated Plants (JKI), Institute for Epidemiology and Pathogen Diagnostics, Messeweg 11-12, 38104 Braunschweig, Germany; 2 Leibniz Institute DSMZ-German Collection of Microorganisms and Cell Cultures, Inhoffenstrasse 7B, 38124 Braunschweig, Germany; 3 Research Institute of Horticulture, Konstytucji 3 Maja 1/3, 96-100, Skierniewice, Poland

## Abstract

Four plant tumorigenic strains 932, 1019, 1078^T^ and 1081 isolated from cane gall tumors on thornless blackberry (*Rubus* sp.) were characterized. They shared low sequence identity with related *Rhizobium* spp. based on comparisons of 16S rRNA gene (≤98%) and housekeeping genes *atpD*, *recA* and *rpoB* (<90%). Phylogenetic analysis indicated that the strains studied represent a novel species within the genus *Rhizobium*, with *Rhizobium tubonense* CCBAU 85046^T^ as their closest relative. Furthermore, obtained average nucleotide identity (ANI) and *in silico* DNA–DNA hybridization (DDH) values calculated for whole-genome sequences of strain 1078^T^ and related *Rhizobium* spp. confirmed the authenticity of the novel species. The ANI-Blast (ANIb), ANI-MUMmer (ANIm) and *in silico* DDH values between strain 1078^T^ and most closely related *R. tubonense* CCBAU 85046^T^ were 76.17%, 84.11% and 21.3%, respectively. The novel species can be distinguished from *R. tubonense* based on phenotypic and chemotaxonomic properties. Here, we demonstrated that four strains studied represent a novel species of the genus *Rhizobium*, for which the name *Rhizobium tumorigenes* sp. nov. is proposed (type strain 1078^T^ = DSM 104880^T^ = CFBP 8567^T^). *R. tumorigenes* is a new plant tumorigenic species carrying the tumor-inducing (Ti) plasmid.

## Introduction

Plant tumorigenic bacteria belonging to the family *Rhizobiaceae* are associated with crown gall and cane gall diseases that can affect various plants^1–3^. The presence of a large conjugal tumor-inducing (Ti) plasmid in the genome of the host strain is essential for pathogenicity. So far, tumorigenic strains have been identified within multiple species of the genus *Agrobacterium*, as well as within species *Allorhizobium vitis* (i.e. *Agrobacterium* biovar 3/*Agrobacterium vitis*) and *Rhizobium rhizogenes* (i.e. *Agrobacterium* biovar 2/*Agrobacterium rhizogenes*).

*Rubus* spp. have been identified as natural hosts of tumorigenic *Rhizobiaceae* strains. Crown gall disease that was mostly associated with tumorigenic strains of *R. rhizogenes* and *A. tumefaciens* species complex (i.e. *Agrobacterium* biovar 1/*Agrobactrium tumefaciens*), including recently described species *Agrobacterium arsenijevicii* has been frequently reported on *Rubus* spp.^4–12^. In general, crown gall disease symptoms include formation of tumors on roots and crowns of infected plants. In addition, tumorigenic *R. rhizogenes* strains were also isolated from aerial tumors formed at pruning wounds of blackberry-raspberry (*Rubus occidentalis*-*Rubus idaeus*) hybrid of cv. Lochness^4^. On the other hand, cane gall disease is characterized by formation of tumors on the cane surface that may increase in size and number and completely girdle affected cane sections in advanced stages of disease^13^. Although *Agrobacterium rubi* was initially recognized as a causal agent of cane gall disease of *Rubus* spp.^13^, later reports on this disease are limited or entirely lacking.

In this study, we observed plants of thornless blackberry (*Rubus* sp.) showing cane gall symptoms corresponding to those described before by Hildebrand^13^, that originated from two plantations in western Serbia. Although disease developed repeatedly every year, it was not lethal for infected blackberry plants nor caused significant losses in yield. Here, we characterized atypical tumorigenic strains isolated from cane gall tumors by using a polyphasic taxonomic approach and demonstrated that they represent a novel tumorigenic species within the genus *Rhizobium*.

## Results and Discussion

Four atypical strains isolated from thornless blackberry showing cane gall symptoms, originating from two localities in western Serbia, were characterized by using polyphasic taxonomic methods. The strains studied possessed identical 16S rRNA gene sequences (calculated for the length of 1309 bp). Furthermore, strains originating from the same locality (932/1019 and 1078^T^/1081) possessed identical sequences of *atpD*, *recA* and *rpoB* housekeeping genes. On the other hand, strains 932 and 1019 had high sequence identities (>97.5%) with strains 1078^T^ and 1081 based on analysis of partial sequences of *atpD* (496 bp), *recA* (541 bp) and *rpoB* (585 bp) housekeeping genes (Table S1), suggesting that they are closely related and belong to the same species. The strains exhibited different PCR MP fingerprints (Fig. S1), which excluded the possibility of their clonal origin. However, strains originating from the same locality showed similar fingerprints, differing by several bands (Fig. S1).

The strains studied shared 16S rRNA gene sequence identity ≤98% with related *Rhizobium* spp. (Table S1). It is notably low value, taking into account 16S rRNA gene sequence identities between related *Rhizobium* species being above 99%, and in some cases even 100%, as it was shown, for example, for *Rhizobium laguerreae* and *Rhizobium leguminosarum*^14^ or *Rhizobium aegyptiacum*, *Rhizobium bangladeshense* and *Rhizobium binae*^15^. Moreover, nucleotide identity values were remarkably low (<90%) when comparing *atpD*, *recA* and *rpoB* gene sequences of novel strains and related species (Table S1).

**Figure 1:**
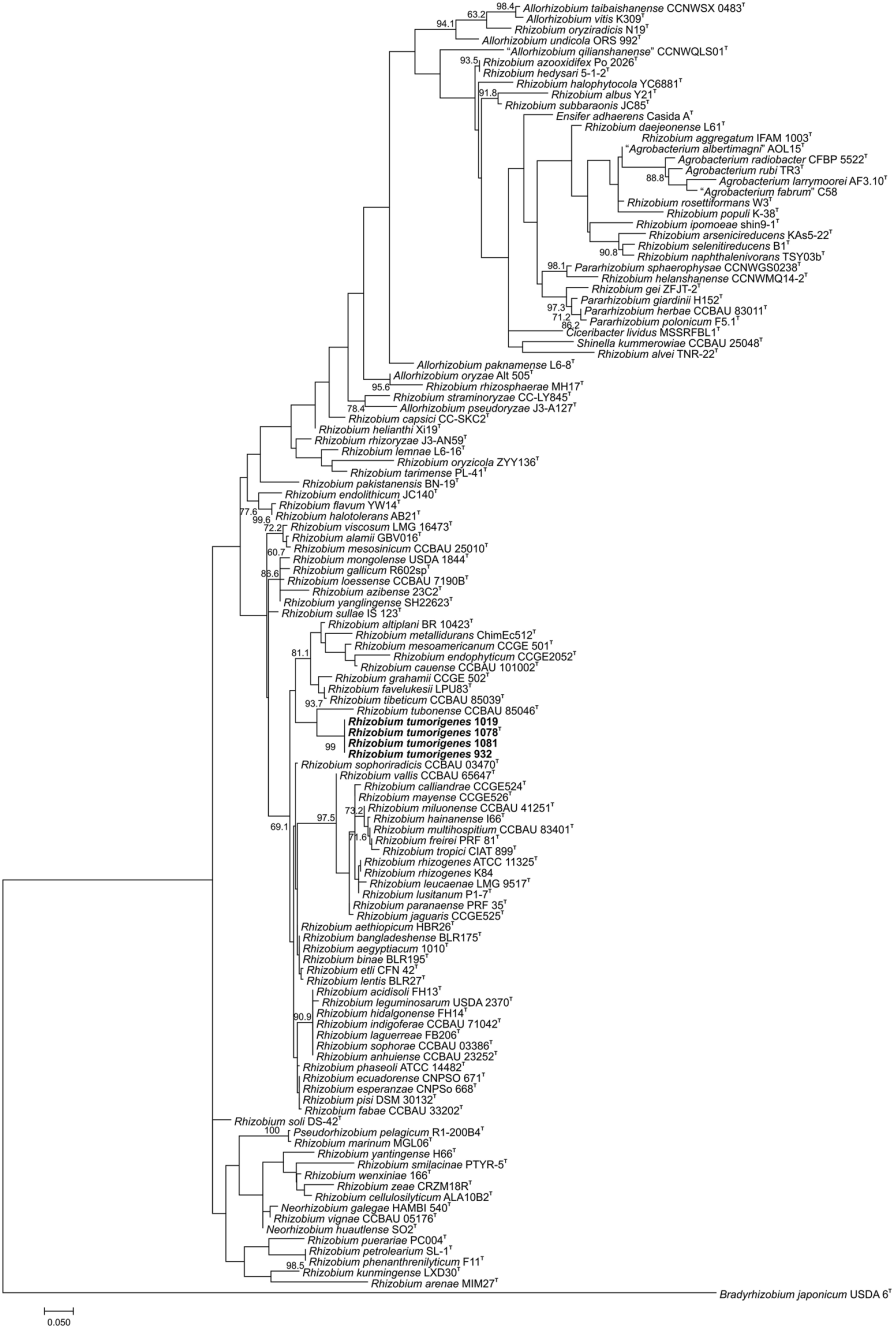
Maximum likelihood tree based on partial sequence of 16S rRNA gene (1273 bp) indicates the phylogenetic position of *Rhizobium tumorigenes* sp. nov. strains 932, 1019, 1078^T^ and 1081 (marked in bold) and their relationship with related members of the *Rhizobiaceae* family. The tree was constructed using a general time reversible substitution model with a gamma distribution and invariant sites (GTR + G + I). Bootstrap values (expressed as a percentage of 1000 replications) equal to or higher than 60% are shown at nodes. *Bradyrhizobium japonicum* USDA 6^T^ was used as the outgroup organism. DDBJ/EMBL/GenBank accession numbers are given in Table S3. The scale bar represents the estimated number of nucleotide substitutions per site.

**Figure 2:**
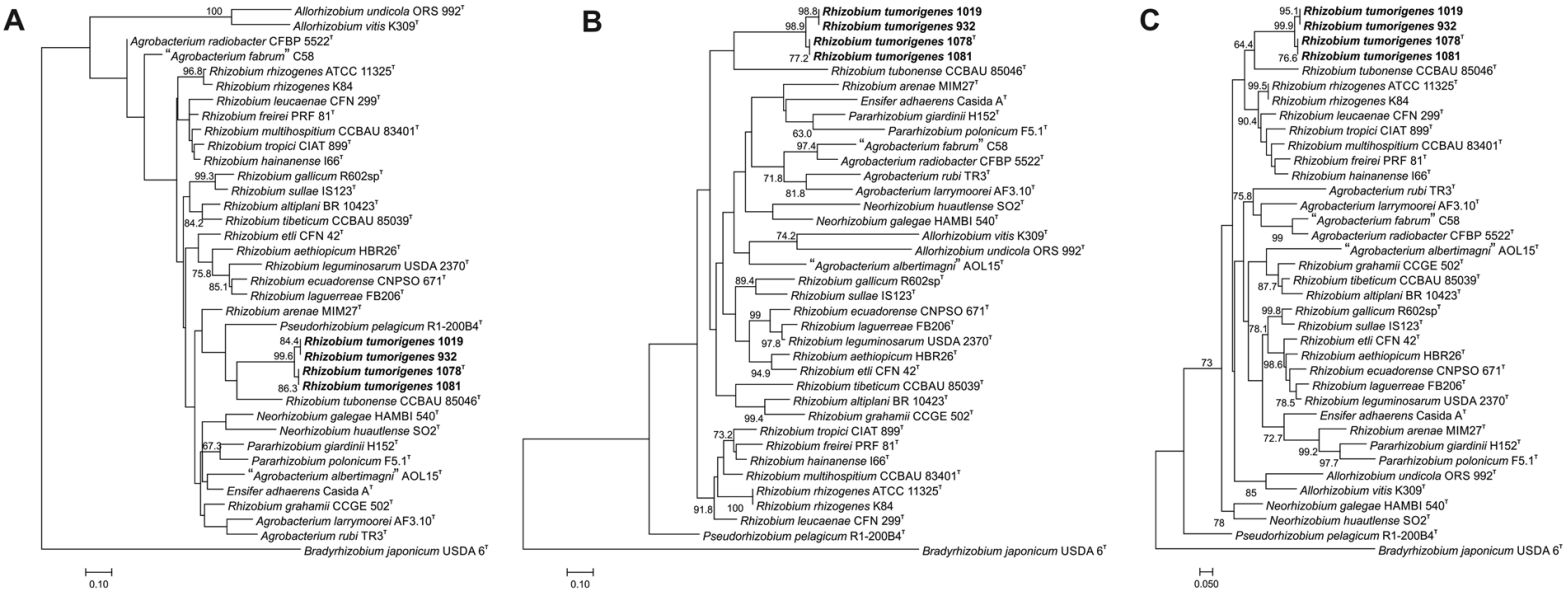
Maximum likelihood trees based on partial sequences of *atpD* – 496 bp (**A**), *recA* – 541 bp (**B**) and *rpoB* – 585 bp (**C**) housekeeping genes indicate the phylogenetic position of *Rhizobium tumorigenes* sp. nov. strains 932, 1019, 1078^T^ and 1081 (marked in bold) and their relationship with related members of the *Rhizobiaceae* family. The trees were constructed using a general time reversible substitution model with a gamma distribution and invariant sites (GTR + G + I). Bootstrap values (expressed as a percentage of 1000 replications) equal to or higher than 60% are shown at nodes. *Bradyrhizobium japonicum* USDA 6^T^ was used as the outgroup organism. DDBJ/EMBL/GenBank accession numbers are shown in Table S3. The scale bar represents the estimated number of nucleotide substitutions per site.

Based on 16S rRNA gene phylogeny, strains studied were grouped within the genus *Rhizobium*, however, they formed a separate cluster, with *Rhizobium tubonense* as their closest relative (Fig. 1). For further phylogenetic analysis, we selected species closely related to novel strains and included representative members of the *Rhizobiaceae* family. Phylogenetic trees generated by using partial sequences of *atpD*, *recA* and *rpoB* genes confirmed independent clustering of the novel strains with *R. tubonense* CCBAU 85046^T^ located on a neighbouring branch (Fig. 2).

The draft genome sequence of *R. tumorigenes* 1078^T^ consisted of 5,899,412 bp (129 contigs) with an average coverage of 127.6x. For *R. tubonense* CCBAU 85046^T^, the assembly generated 85 contigs comprising of 6,540,512 bp with an average coverage of 131.8x. *R. tumorigenes* 1078^T^ and *R. tubonense* CCBAU 85046^T^ had similar average GC contents of 60.0% and 59.3%, respectively, which was generally in accordance with other related *Rhizobium* spp., e.g. *R. rhizogenes* ATCC 11325^T^ (59.9%), *Rhizobium tropici* CIAT 899^T^ (59.9%) or *Rhizobium freirei* PRF 81^T^ (59.9%).

**Figure 3:**
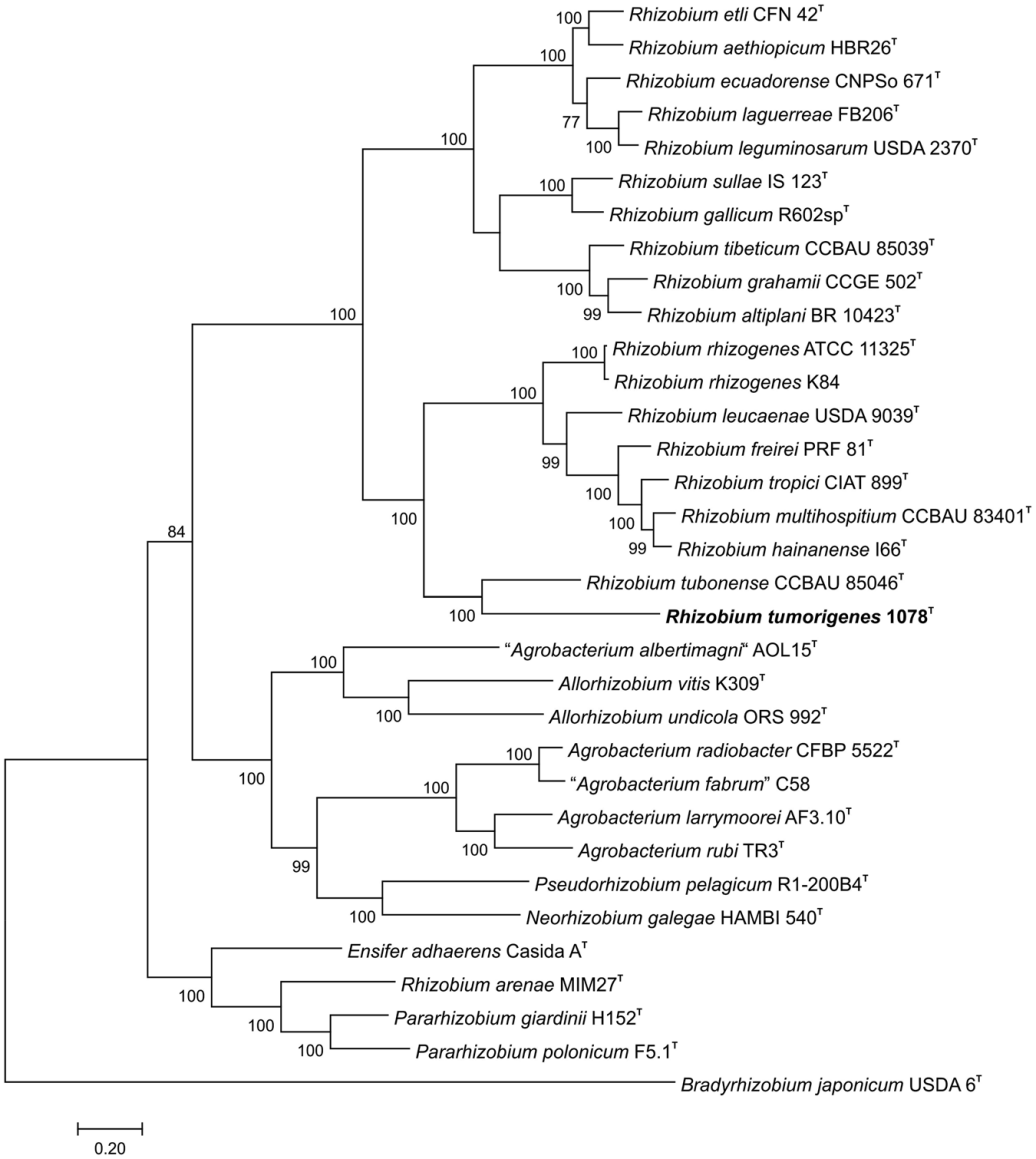
Maximum likelihood phylogenomic tree based on concatenated sequences of 385 conserved proteins extracted from whole-genome sequences showing the evolutionary relationships between *Rhizobium tumorigenes* sp. nov. 1078^T^ (marked in bold) and related *Rhizobiaceae* members. Branch support values equal to or higher than 60% are shown at nodes. *Bradyrhizobium japonicum* USDA 6^T^ was used as the outgroup organism. DDBJ/EMBL/GenBank whole-genome accession numbers are shown in Table S3. The scale bar represents the estimated number of amino acid substitutions per site.

Genome-wide phylogeny based on 385 conserved proteins further supported distinctiveness of representative strain 1078^T^ and its phylogenetic relationship to *R. tubonense* CCBAU 85046^T^ (Fig. 3). Furthermore, whole-genome sequences of strain 1078^T^ and related *Rhizobium* spp. were compared by using ANI-Blast (ANIb), ANI-MUMmer (ANIm) and *in silico* DDH methods. Obtained values were far below the proposed threshold for species delineation, which ranges between 95–96% for ANI^16^ or is 70% for DDH^17–19^, confirming the authenticity of the novel species (Table 1). The ANIb, ANIm and *in silico* DDH values between strain 1078^T^ and most closely related *R. tubonense* CCBAU 85046^T^ were 76.17%, 84.11% and 21.3%, respectively. In case of ANIm, less than 20% of the genome was aligned for all strains used for comparison, and the alignment was assigned by the software as suspicious. However, besides other strains when it was below 15%, almost 20% (19.11%) of the genome was aligned when strain 1078^T^ was compared with *R. tubonense* CCBAU 85046^T^, which is a borderline for reliable alignment. Although evidently distantly related, *R. tubonense* CCBAU 85046^T^ was considered as a closest known relative of novel strains isolated from blackberry, with respect to their phylogenetic, phylogenomic and genomic relatedness. Therefore, phenotypic and chemotaxonomic characterization was performed in order to determine additional traits distinguishing these two species.

**Table 1:** Average nucleotide identity (ANI) and *in silico* DNA–DNA hybridization (DDH) comparisons between *Rhizobium tumorigenes* sp. nov.

Species	Strain	Accession Numbers^a^	ANI values (%)		*in silico* DDH (%)
			ANIb^b^	ANIm^c^	
*Rhizobium tubonense*	CCBAU 85046^T^	PCDP01	76.15	84.03^d^	21.3
*Rhizobium rhizogenes*	ATCC 11325^T^	BAYX01	75.7	83.87^d^	21
*Rhizobium rhizogenes*	K84	CP000628, CP000629	75.65	83.76^d^	20.9
*Rhizobium tropici*	CIAT 899^T^	CP004015	75.53	83.76^d^	21
*Rhizobium freirei*	PRF 81^T^	AQHN01	75.24	83.71^d^	21.1
*Rhizobium leucaenae*	USDA 9039^T^	AUFB01	75.24	83.76^d^	21
*Rhizobium multihospitium*	CCBAU 83401^T^	FMAG01	75.16	83.72^d^	20.7
*Rhizobium hainanense*	I66^T^	FMAC01	75.06	83.69^d^	20.8
*Rhizobium ecuadorense*	CNPSo 671^T^	LFIO01	75.04	83.94^d^	20.7
*Rhizobium laguerreae*	FB206^T^	MRDM01	74.94	83.89^d^	20.7
*Rhizobium leguminosarum*	USDA 2370^T^	MRDL01	74.91	83.85^d^	20.7
*Rhizobium etli*	CFN 42^T^	CP000133	74.8	83.82^d^	20.6
*Rhizobium aethiopicum*	HBR26^T^	FMAJ01	74.68	83.62^d^	20.5

1078^T^ (GenBank accession no. PCDQ01) and related *Rhizobium* spp. ^a^Accession numbers refer to draft genomes or chromosome sequences.

^b^ANI-Blast.

^c^ANI-MUMmer.

^d^Less than 20% of the genome has been aligned.

The results of phenotypic characterization of novel strains are summarized in Table 2. Unlike *R. tubonense* CCBAU 85046^T^, the novel strains from blackberry were able to catabolize L-Alanine and D-Gluconic acid. On the other hand, *R. tubonense* CCBAU 85046^T^ utilized L-Lactic acid, contrary to the novel strains studied. However, many genes encoding transport and catabolism of carbon and nitrogen compounds can be plasmid-borne, and therefore, the role of phenotypic tests in taxonomy of *Rhizobium* spp. has been recently called into question^20^. Moreover, biochemical tests were of limited value for classification and differentiation of some *Rhizobiaceae* species as indicated by Puławska, *et al*.^21^.

**Table 2:** Protologue for *Rhizobium tumorigenes* sp. nov.

Taxonumber	TA00285
Species name	*Rhizobium tumorigenes*
Genus name	*Rhizobium*
Specific epithet	*tumorigenes*
Species status	sp. nov.
Species etymology	tu,mo.ri’ge.nes. L. masc. n. tumor swelling, tumor; N.L. suff. genes (from Gr. v. gennaô, to produce), producing; N.L. part. adj. tumorigenes tumor-producing
Designation of the type strain	1078
Strain collection numbers	DSM 104880 = CFBP 8567
16S rRNA gene accession number	MG018989
Alternative housekeeping genes	*atpD* [MG007664], *recA* [MG007669], *repB* [MG007674]
Genome accession number	PCDQ00000000
Genome status	draft
Genome size	5899.41 kbp
GC mol %	60.0
Country of origin	Serbia
Region of origin	Arilje Municipality, Zlatibor District
Date of isolation	2016
Source of isolation	Cane gall tumors on thornless blackberry (*Rubus* sp.)
Sampling date	2016
Number of strains in study	4
Source of isolation of non-type strains	Cane gall tumors on thornless blackberry (*Rubus* sp.)
Growth medium, incubation conditions used for standard cultivation	Yeast mannitol agar (YMA) at 22 °C
Conditions of preservation	−80 °C
Gram stain	Negative
Cell shape	Rod
Colony morphology	Colonies on YMA are white to cream coloured, circular, convex and glistening
Positive tests with BIOLOG	Dextrin, D-Maltose, D-Trehalose, D-Cellobiose, Gentiobiose, Sucrose, D-Turanose, pH 6, α-D-Lactose, D-Melibiose, N-Acetyl-D-Glucosamine, N-Acetyl-β-D-Mannosamine, α-D-Glucose, D-Mannose, D-Fructose, D-Galactose, D-Sorbitol, D-Mannitol, D-Arabitol, myo-Inositol, Glycerol, Troleandomycin, Rifamycin SV, L-Alanine, L-Glutamic Acid, Lincomycin, Pectin, D-Gluconic Acid, Tetrazolium Blue, L-Malic Acid, Bromo-Succinic Acid, Tween 40, Acetoacetic Acid
Negative tests with BIOLOG	Stachyose, pH5, N-Acetyl-D-Galactosamine, N-Acetyl Neuraminic Acid, 4% NaCl, 8% NaCl, Inosine, Fusidic Acid, D-Serine (sensitivity assay), D-Aspartic Acid, D-Serine, Minocycline, L-Arginine, L-Aspartic Acid, L-Pyroglutamic Acid, Guanidine HCl, Niaproof 4, Quinic Acid, D-Saccharic Acid, p-Hydroxy-Phenylacetic Acid, L-Lactic Acid, Lithium Chloride, α-Hydroxy-Butyric Acid, β-Hydroxy-D,L-butyric Acid, α-Keto-Butyric Acid, Propionic Acid, Formic Acid, Sodium Butyrate, Sodium Bromate
Positive tests with API	URE, ESC, PNG, GLU (assimilation), ARA, MNE, MAN, NAG, MAL, MLT
Negative tests with API	NO3, TRP, GLU (fermentation), ADH, GEL, CAP, ADI, PAC
Variable tests with API	GNT, CIT
Commercial kits used	BIOLOG GEN3, API 20NE
Major fatty acids	18:1 w7c (66.11–70.93%), 19:0 cyclo w8c (8.71–12.40%), Summed feature 2 (12:0 aldehyde?, unknown fatty acid of ECL 10.928, 16:1 iso I/14:0 3OH; 5.88–6.23%) and 16:0 (4.07–5.63%)
Known pathogenicity	Plant pathogenic

The major cellular fatty acids of the four novel strains were: 18:1 w7c (66.11–70.93%), 19:0 cyclo w8c (8.71–12.40%), Summed feature 2 (12:0 aldehyde and/or an unknown fatty acid of equivalent chain length 10.928, and 14:0 3OH/16:1 iso I; 5.88–6.23%) and 16:0 (4.07–5.63%) (Table S2). Comparing to four strains studied, *R. tubonense* CCBAU 85046^T^ possessed a lower content of fatty acid 18:1 w7c (55.11%), and a higher one of 16:0 (10.65%) and 11 methyl 18:1 w7c (6.72%) (Table S2).

By using PCR, presence of *virC*, *virD2*, *ipt* and *tms2* genes was detected in all four strains studied, indicating that they carry the Ti plasmid required for plant tumorigenic ability. In pathogenicity assay, all strains caused tumors on inoculated sunflower seedlings and kalanchoe plants. In contrast to strains 1078^T^ and 1081 which clearly induced tumors on kalanchoe stems, tumors induced by strains 932 and 1019 were smaller, which could suggest differences in the virulence of the strains. In case of tomato, the reaction of plants was variable, since strains caused either very small and inconspicuous tumors, or symptom development was absent.

Overall, based on the polyphasic characterization of the four strains isolated from cane gall tumors on thornless blackberry, we propose that they represent a novel species, *Rhizobium tumorigenes* sp. nov., with 1078^T^ (=DSM 104880^T^ = CFBP 8567^T^) as the type strain. *R. tumorigenes* sp. nov. is a new plant tumorigenic species containing the Ti plasmid and the second tumorigenic species within the genus *Rhizobium*. Tumor-inducing ability has been limited so far to *Agrobacterium* spp., *A. vitis* and *R. rhizogenes*.

The new species is registered at Digital Protologue website the (http://imedea.uib-csic.es/dprotologue/) under the taxonumber TA00285. The description of the new species is given in Table 2.

## Materials and Methods

### Bacterial strains and DNA extraction

Four strains 932 (=DSM 104878 = CFBP 8566), 1019 (=DSM 104919), 1078^T^ (=DSM 104880^T^ = CFBP 8567^T^) and 1081 (=DSM 104920) recovered from tumor tissue on thornless blackberry (*Rubus* sp.), cultivar ‘Čačak Thornless’ were characterized in this study. They were isolated on yeast mannitol agar (YMA)^12^ from plant samples originating from two localities, Lučani (932 and 1019) and Arilje (1078^T^ and 1081), in western Serbia during 2015–2016. In addition, we also included type strain of *R. tubonense* CCBAU 85046^T^ for some tests. For molecular methods, total genomic DNA of the strains was extracted from bacteria grown on YMA at 22 °C for 48 h according to the procedure described by Aljanabi and Martinez^22^.

### PCR melting profile (PCR MP) fingerprinting

Genetic diversity among four novel strains was investigated by a method of PCR melting profile (PCR MP) with two sets of restriction enzymes, adaptors and primers: *Apa*I and *Hin*dIII as described by Puławska, *et al*.^23^. Denaturation temperatures 91 °C and 89 °C were used for PCR MP with *Apa*I and *Hin*dIII enzymes, respectively.

### PCR amplification and sequencing of 16S rRNA and housekeeping genes

The amplification and sequencing of nearly complete 16S rRNA gene was performed by using fD1 and rP2 primers^24^, as described by Kuzmanović, *et al*.^12^. Primer sets atpD-273F/771R^25^, and rpoB-456F/1061R^26^ were used for amplification and sequencing of *atpD* and *rpoB* gene fragments, respectively. PCR reactions were performed in a 25 µl volume with master mix containing 1 × Colourless GoTaq Flexi buffer (Promega Corp., USA), 1.5 mmol l^−1^ MgCl_2_, 0.2 mmol l^−1^ of each dNTP, 0.2 µmol l^−1^ of each primer, 0.5 U of GoTaq Flexi DNA polymerase (Promega Corp., USA) and 40–60 ng of DNA template. The thermal profile for amplification of *atpD* gene fragment was as described by Gaunt, *et al*.^25^, except that total of 35 cycles with annealing temperature of 60 °C, followed by final extension at 72 °C for 5 min were used. For amplification of *rpoB* gene fragment, the PCR conditions were as follows: initial denaturation at 95 °C for 5 min; 35 cycles of denaturation at 94 °C for 1 min, annealing at 58 °C for 1 min and extension at 72 °C for 1 min. A final extension at 72 °C for 5 min was conducted. The amplification and sequencing of *recA* gene fragment was performed by using primers F2898/F2899^27^, as described before^12^.

### Gene sequence comparison and phylogenetic analysis

The phylogenetic analysis and sequence comparisons were conducted on 16S rRNA gene sequence, and sequences of *atpD*, *recA* and *rpoB* housekeeping genes. Sequences of related *Rhizobiaceae* strains were retrieved from NCBI GenBank and included into the analysis (Table S3). The obtained sequences were aligned using MUSCLE^28^ at EMBL-EBI^29^.

Pairwise nucleotide identities were calculated using the p-distance model with MEGA 7.0.21 software package^30^. Maximum likelihood (ML) trees were generated with PhyML 3.0^31^ by using 1000 bootstrap replicates. The most suitable substitution models were determined by the Smart Model Selection (SMS) tool^32^ and jModelTest 2.1.10^33^, according to the Akaike information criterion (AIC)^34^.

### Genome sequencing

DNA fragmentation was performed on Covaris E210 and libraries were made with NEBNext DNA Library Prep Master Mix Set for Illumina® (NEB, USA). Sequencing was performed on Illumina MiSeq platform using MiSeq Reagent Kit v2 (500-cycles) in PE250 mode generating 3,336,198 (1078^T^) and 3,784,696 (*R. tubonense* CCBAU 85046^T^) sequences in pairs (Genomed SA, Poland). Sequence processing and assembly were performed using CLC Genomics Workbench 7.5.

### Whole-genome sequence comparisons and phylogenomic analysis

Genome sequence of strain 1078^T^ was compared with genome sequences of related *Rhizobium* spp., by calculating average nucleotide identity (ANI) values using the JSpecies Web Service^35^. *In silico* DNA–DNA hybridizations (DDH) values by the Genome-to-GenomeDistance Calculator (GGDC 2.1; http://ggdc.dsmz.de/distcalc2.php) using the recommended BLAST + alignment and formula 2 (identities/HSP length)^17^ were also obtained.

Genome-wide phylogeny based on 385 conserved protein sequences extracted from genome sequences of 1078^T^ and strains of related *Rhizobiaceae* strains was reconstructed by using PhyloPhlAn pipeline, version 0.99^36^.

### Phenotypic characterization

Novel strains isolated from blackberry, including *R. tubonense* CCBAU 85046^T^ were phenotypically characterized by using API and Biolog tests. The API 20NE kit was used according to manufacturer’s instructions (bioMérieux) and addition of MgSO_4_ in order to improve bacterial growth as described before by Saidi, *et al*.^14^. Utilization of sole carbon sources was tested with Biolog GEN III microplates by using protocol C2 according to the instructions of the manufacturer (Biolog, Inc., Hayward, CA, USA). Measurements were taken after incubation of API strips and Biolog microplates at 20 °C for 72 h.

### Chemotaxonomic analysis

Analysis of cellular fatty acid composition of the novel strains isolated from blackberry, including *R. tubonense* CCBAU 85046^T^ was performed by the Microbial Identification System (Sherlock version 6.1, TSBA40 method), as recommended by the manufacturer. Since the bacteria did not grow well on standard trypticase soy agar (TSA) medium, they were cultured on YMA at 22 °C for 36 h.

### Detection of tumor-inducing (Ti) plasmid and pathogenicity assay

Bacterial strains isolated from blackberry were subjected to PCR analysis using primers specific for tumor-inducing (Ti) plasmid genes: *virC* (VCF3/VCR3)^37^, *virD2* (A/C’) and *ipt* (CYT/CYT’)^38^, and *tms2* (tms2F1/tms2R2)^39^, as described before^12^.

Pathogenicity of the novel strains originating from Serbia was studied by inoculating stem internodes of young tomato (*Solanum lycopersicum*) and kalanchoe (*Kalanchoe daigremontiana*) plants, and hypocotyls of sunflower (*Helianthus annuus*) seedlings, as described before^40^.

### Accession numbers

The DDBJ/EMBL/GenBank accession numbers for the partial 16S rRNA gene sequences of the strains 1081, 1078^T^, 1019 and 932 are MG018988-MG018991, respectively. Accession numbers for the partial *atpD* gene sequences of the strains *R. tubonense* CCBAU 85046^T^, 1019, 1078^T^, 1081 and 932 are MG007662-MG007666, respectively. Accession numbers for the partial *recA* gene sequences of the strains of the strains *R. tubonense* CCBAU 85046^T^, 1019, 1078^T^, 1081 and 932 are MG007667-MG007671, respectively. Accession numbers for the partial *rpoB* gene sequences of the strains *R. tubonense* CCBAU 85046^T^, 1019, 1078^T^, 1081 and 932 are MG007672-MG007676, respectively.

The draft genome sequences of *R. tumorigenes* 1078^T^ and *R. tubonense* CCBAU 85046^T^ have been deposited at DDBJ/EMBL/GenBank under the accession numbers PCDQ00000000 and PCDP00000000, respectively.

## Electronic supplementary material

Supplementary Dataset
