# Supplementary material for: Rhizobium tumorigenes sp. nov., a novel plant tumorigenic bacterium isolated from cane gall tumors on thornless blackberry
Source: Sci Rep. 2018 Jun 13;8:9051. doi: 10.1038/s41598-018-27485-z (PMC5998023; doi:10.1038/s41598-018-27485-z)

Supplementary Information

Rhizobium tumorigenes sp. nov., a novel plant tumorigenic bacterium isolated from cane gall tumors on thornless blackberry

Nemanja Kuzmanović[1,*], Kornelia Smalla[1], Sabine Gronow[2], Joanna Puławska[3]

[1]Julius Kühn-Institut, Federal Research Centre for Cultivated Plants (JKI), Institute for Epidemiology and Pathogen Diagnostics, Messeweg 11-12, 38104 Braunschweig, Germany
[2]Leibniz Institute DSMZ-German Collection of Microorganisms and Cell Cultures, Inhoffenstrasse 7B, 38124 Braunschweig, Germany
[3]Research Institute of Horticulture, Konstytucji 3 Maja 1/3, 96-100 Skierniewice, Poland
*kuzmanovic1306@gmail.com

* Correspondence and requests for materials should be addressed to NK (e-mail: kuzmanovic1306@gmail.com)

Table S1. Pairwise nucleotide identities (%) between *Rhizobium tumorigenes* sp. nov. 1078^{T} and related *Rhizobium* spp. calculated for partial sequences of 16S rRNA gene (1273 bp) and housekeeping genes *atpD* (496 bp), *recA* (541 bp) and *rpoB* (585 bp).

Species	Strain	16S rRNA	Housekeeping genes		
			atpD	*recA*	*rpoB*
Rhizobium tumorigenes	932	100	98.4	97.6	98.1
Rhizobium tubonense	CCBAU 85046^{T}	97.8	88.3	86.7	86.5
Rhizobium rhizogenes	ATCC 11325^{T}	97.6	87.9	87.1	87.9
Rhizobium rhizogenes	K84	97.6	86.9	87.1	87.9
Rhizobium tropici	CIAT 899^{T}	97.0	87.5	86.9	86.5
Rhizobium freirei	PRF 81^{T}	97.3	88.7	88.0	86.2
Rhizobium leucaenae	USDA 9039^{T}	97.4	86.5	88.7	87.9
Rhizobium multihospitium	CCBAU 83401^{T}	97.4	86.9	87.6	85.8
Rhizobium hainanense	I66^{T}	97.4	87.5	87.8	87.5
Rhizobium ecuadorense	CNPSo 671^{T}	97.6	86.9	85.8	85.3
Rhizobium laguerreae	FB206^{T}	98.0	87.9	85.0	84.8
Rhizobium leguminosarum	USDA 2370^{T}	97.8	87.5	86.3	84.8
Rhizobium etli	CFN 42^{T}	97.6	86.9	86.7	83.9
Rhizobium aethiopicum	HBR26^{T}	97.9	87.1	87.1	85.5

Table S2. Fatty acid composition of *Rhizobium tumorigenes* sp. nov. and its closest relative *Rhizobium tubonense*.

Fatty acid[a]	*Rhizobium tumorigenes* sp. nov.				*Rhizobium tubonense*
	932	1019	1078[T]	1081	CCBAU 85046[T]
13:1 AT 12–13	0.53		0.48	0.55	0.40
16:0	5.63	5.63	4.27	4.07	10.65
16:0 3OH	2.37	2.13	2.02	1.84	2.12
16:1 w11c					0.48
18:0	1.65	1.74	2.18	2.17	2.14
18:0 3OH	1.46	1.22	1.52	1.43	1.23
18:1 2OH					1.52
18:1 w7c	66.11	68.18	70.93	70.73	55.11
18:1 w9c			0.48		0.61
11 methyl 18:1 w7c	2.73	2.77	2.33	3.06	6.72
19:0 cyclo w8c	12.40	11.58	8.71	9.18	9.68
20:2 w6,9c	0.57				
Summed feature 2	5.88	5.92	6.23	6.19	7.11
Summed feature 3					1.17
unknown 14.959	0.67	0.83	0.85	0.80	1.07

[a] Summed features are groups of two or three fatty acids that cannot be separated by GLC with the MIDI system. Summed feature 2: 12:0 aldehyde?, unknown fatty acid of ECL 10.928, 16:1 iso I/14:0 3OH; Summed feature 3: 16:1 w7c/15:0 iso 2OH.

Table S3. The GenBank/EMBL/DDBJ accession numbers for the nucleotide sequences used in phylogenetic analysis. Accession numbers reported in this study are shown in bold.

Strains	16S rRNA	*atpD*	*recA*	*rpoB*	Whole-genome[a]
Rhizobium tumorigenes 932	**MG018991**	**MG007666**	**MG007671**	**MG007676**	
Rhizobium tumorigenes 1019	**MG018990**	**MG007663**	**MG007668**	**MG007673**	
Rhizobium tumorigenes 1078[T]	**MG018989**	**MG007664**	**MG007669**	**MG007674**	**PCDQ01**
Rhizobium tumorigenes 1081	**MG018988**	**MG007665**	**MG007670**	**MG007675**	
Rhizobium tubonense CCBAU 85046[T]	EU256434	**MG007662**	**MG007667**	**MG007672**	**PCDP01**
"*Agrobacterium albertimagni*" AOL15[T]	ALJF01000029	ALJF01000002	ALJF01000008	ALJF01000027	ALJF01
"*Agrobacterium fabrum*" C58	AE007869	AE007869	AE007869	AE007869	AE007869- AE007872
Agrobacterium larrymoorei AF3.10[T]	Z30542	HQ735088	JADW01000018	FR754360	JADW01
Agrobacterium radiobacter CFBP 5522[T]	AB247615	KF206563	LMVJ01000001	KF206906	LMVJ01
Agrobacterium rubi TR3[T]	X67228	KF206562	BBJU01000013	KF206905	BBJU01
Allorhizobium oryzae Alt 505[T]	EU056823				
Allorhizobium paknamense L6-8[T]	AB733647				
Allorhizobium pseudoryzae J3-A127[T]	DQ454123				
"*Allorhizobium qilianshanense*" CCNWQLS01[T]	NR_132606				
Allorhizobium taibaishanense CCNWSX 0483[T]	HM776997				
Allorhizobium undicola ORS 992[T]	Y17047	JHXQ01000004	JHXQ01000002	JHXQ01000031	JHXQ01
Allorhizobium vitis K309[T]	U45329	LMVL01000034	FR847962	KF206929	LMVL01
Bradyrhizobium japonicum USDA 6[T]	U69638	AP012206	AP012206	AP012206	NC_017249
Ciceribacter lividus MSSRFBL1[T]	JQ230000				
Ensifer adhaerens Casida A[T]	AM181733	JNAE01000159	JNAE01000117	JNAE01000120	JNAE01
Neorhizobium galegae HAMBI 540[T]	AB680726	KF206641	HG938353	KF206983	HG938353, HG938354
Neorhizobium huautlense SO2[T]	NR_024863	KF206569	KF206825	KF206912	
Pararhizobium herbae CCBAU 83011[T]	GU565534				
Pararhizobium giardinii H152[T]	EU488750	KF206567	ARBG01000193	ARBG01000175	ARBG01
Pararhizobium polonicum F5.1[T]	LGLV01000030	LGLV01000010	LGLV01000020	LGLV01000028	LGLV01
Pararhizobium sphaerophysae CCNWGS0238[T]	FJ154088				
Pseudorhizobium pelagicum R1-200B4[T]	JOKI01000038	JOKI01000007	JOKJ01000001	JOKJ01000051	JOKJ01
Rhizobium acidisoli FH13[T]	KJ921033				
Rhizobium aegyptiacum 1010[T]	NR_137399				
Rhizobium aethiopicum HBR26[T]	FMAJ01000055	FMAJ01000010	FMAJ01000001	FMAJ01000038	FMAJ01
Rhizobium aggregatum IFAM 1003[T]	X73041				
Rhizobium alamii GBV016[T]	AM931436				

Rhizobium albus Y21^T	KU245743				
Rhizobium altiplani BR 10423^T	LNCD01000123	LNCD01000123	LNCD01000082	LNCD01000061	LNCD01
Rhizobium alvei TNR-22^T	HE649224				
Rhizobium anhuiense CCBAU 23252^T	NR_137229				
Rhizobium arenae MIM27^T	KX362244	KY047782	KY047781	MOOY01000001	MOOY01
Rhizobium arsenicireducens KAs5-22^T	JX173993				
Rhizobium azibense 23C2^T	NR_133841				
Rhizobium azooxidifex Po 20/26^T	LN832063				
Rhizobium bangladeshense BLR175^T	NR_137241				
Rhizobium binae BLR195^T	NR_137242				
Rhizobium calliandrae CCGE524^T	JX855162				
Rhizobium capsici CC-SKC2^T	NR_136447				
Rhizobium cauense CCBAU 101002^T	NR_133049				
Rhizobium cellulosilyticum ALA10B2^T	DQ855276				
Rhizobium daejeonense NBRC 102495^T	AB681832				
Rhizobium ecuadorense CNPSO 671^T	LFIO01000095	LFIO01001065	LFIO01000381	LFIO01001107	LFIO01
Rhizobium endolithicum JC140^T	HE818072				
Rhizobium endophyticum CCGE2052^T	EU867317				
Rhizobium esperanzae CNPSo 668^T	JN129376; MXPU01000002				
Rhizobium etli CFN 42^T	U28916	NC_007761	NC_007761	NC_007761	CP000133
Rhizobium fabae CCBAU 33202^T	DQ835306				
Rhizobium favelukesii LPU83^T	HG916852				
Rhizobium flavum YW14^T	NR_133843				
Rhizobium freirei PRF 81^T	EU488742	AQHN01000005	AQHN01000010	AQHN01000082	AQHN01
Rhizobium gallicum R602sp^T	U86343	CP006877	CP006877	CP006877	CP006877- CP006880
Rhizobium gei ZFJT-2^T	KF551166				
Rhizobium grahamii CCGE 502^T	JF424608	JF424612	AEYE02000013	AEYE02000009	AEYE02
Rhizobium hainanense I66^T	U71078	KF206565	FMAC01000001	FMAC01000028	FMAC01
Rhizobium halophytocola YC6881^T	GU322905				
Rhizobium halotolerans AB21^T	JX307098				
Rhizobium hedysari 5-1-2^T	KU358685				
Rhizobium helanshanense CCNWQTX14^T	HQ132351				
Rhizobium helianthi Xi19^T	JQ032629				
Rhizobium hidalgonense FH14^T	KJ921034				
Rhizobium indigoferae CCBAU 71042^T	AY034027				
Rhizobium ipomoeae shin9-1^T	HE866935				
Rhizobium jaguaris CCGE525^T	JX855169				

Rhizobium laguerreae FB206^T	JN558651	MRDM01000011	MRDM01000014	MRDM01000033	MRDM01
Rhizobium leguminosarum USDA 2370^T	U29386	MRDL01000015	MRDL01000051	MRDL01000032	MRDL01
Rhizobium lemnae L6-16^T	NR_126174				
Rhizobium lentis BLR27^T	NR_137243				
Rhizobium leucaenae CFN 299^T	X67234	AUFB01000004	AUFB01000010	AUFB01000052	AUFB01
Rhizobium loessense CCBAU 7190B^T	AF364069				
Rhizobium lusitanum P1-7^T	AY738130				
Rhizobium kunmingense LXD30^T	NR_132597				
Rhizobium marinum MGL06^T	KJ751545				
Rhizobium mayense CCGE526^T	JX855172				
Rhizobium mesoamericanum CCGE 501^T	JF424606				
Rhizobium mesosinicum CCBAU 25010^T	DQ100063				
Rhizobium metallidurans ChimEc512^T	JX678769				
Rhizobium miluonense CCBAU 41251^T	EF061096				
Rhizobium mongolense USDA 1844^T	U89817				
Rhizobium multihospitium CCBAU 83401^T	EF035074	FMAG01000009	FMAG01000007	FMAG01000018	FMAG01
Rhizobium naphthalenivorans TSY03b^T	AB663504				
Rhizobium oryzicola ZYY136^T	NR_137225				
Rhizobium oryziradicis N19^T	KX129901				
Rhizobium pakistanensis BN-19^T	AB854065				
Rhizobium paranaense PRF 35^T	NR_134152				
Rhizobium petrolearium SL-1^T	EU556969				
Rhizobium phaseoli ATCC 14482^T	EF141340				
Rhizobium phenanthrenilyticum F11^T	FJ743436				
Rhizobium pisi DSM 30132^T	AY509899				
Rhizobium populi K-38^T	NR_134153				
Rhizobium puerariae PC004^T	LC014930				
Rhizobium rhizogenes ATCC 11325^T	AY945955	BAYX01000004	BAYX01000001	BAYX01000002	BAYX01
Rhizobium rhizogenes K84	CP000628	CP000628	CP000628	CP000628	CP000628-CP000632
Rhizobium rhizoryzae J3-AN59^T	NR_133844				
Rhizobium rhizosphaerae MH17^T	KX129902				
Rhizobium rosettiformans w3^T	EU781656				
Rhizobium selenitireducens B1^T	EF440185				
Rhizobium smilacinae PTYR-5^T	KF551141				
Rhizobium soli DS-42^T	EF363715				
Rhizobium sophorae CCBAU 03386^T	KJ831229				
Rhizobium sophoriradicis CCBAU 03470^T	KJ831225				

Rhizobium straminoryzae CC-LY845^{T}	NR_126295				
Rhizobium subbaraonis JC85^{T}	FR714938				
Rhizobium sullae IS 123^{T}	Y10170	FWER01000018	FWER01000058	FWER01000079	FWER01
Rhizobium tarimense PL-41^{T}	HM371420				
Rhizobium tibeticum CCBAU 85039^{T}	EU256404	KF206621	FNXB01000016	FNXB01000071	FNXB01
Rhizobium tropici CIAT 899^{T}	U89832	CP004015	CP004015	CP004015	CP004015
Rhizobium vallis CCBAU 65647^{T}	FJ839677				
Rhizobium vignae CCBAU 05176^{T}	GU128881				
Rhizobium viscosum LMG 16473^{T}	NR 042253				
Rhizobium wenxiniae 166^{T}	KR610521				
Rhizobium yanglingense SH22623^{T}	AF003375				
Rhizobium yantingense H66^{T}	KC934840				
Rhizobium zeae CRZM18R^{T}	KX932068				
Shinella kummerowiae CCBAU 25048^{T}	EF070131				

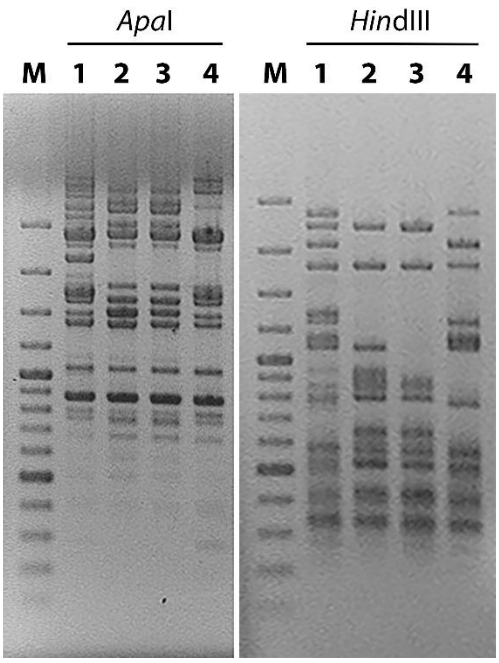

Fig. S1. Electrophoretic patterns obtained after PCR MP with enzymes *Apa*I and *Hin*dIII. 1 – 1081, 2 – 932, 3 – 1019, 4 – 1078^{T}, M – marker 100 bp Ladder (Thermo Scientific Fermentas, #SM0321). Original gel photos are shown below.

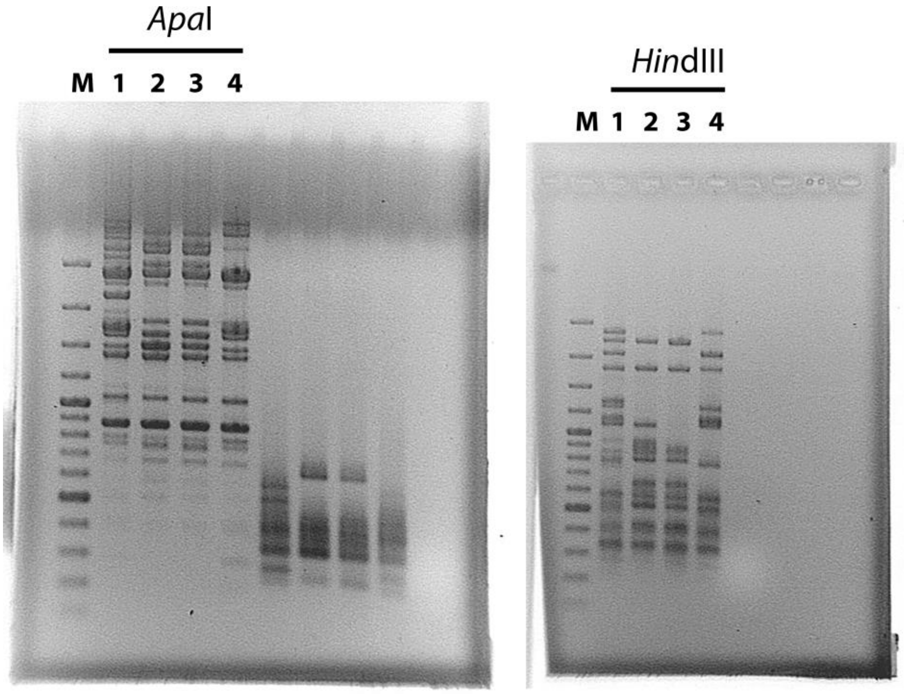

Supplement: Supplementary file 1 — Supplementary Dataset [file 41598_2018_27485_MOESM1_ESM.pdf]
